# Marine Biosurfactants: Biosynthesis, Structural Diversity and Biotechnological Applications

**DOI:** 10.3390/md17070408

**Published:** 2019-07-09

**Authors:** Sonja Kubicki, Alexander Bollinger, Nadine Katzke, Karl-Erich Jaeger, Anita Loeschcke, Stephan Thies

**Affiliations:** 1 Institute of Molecular Enzyme Technology, Heinrich-Heine-University Düsseldorf, Forschungszentrum Jülich, D-52425 Jülich, Germany; 2 Institute of Bio- and Geosciences IBG-1: Biotechnology, Forschungszentrum Jülich GmbH, D-52425 Jülich, Germany

**Keywords:** marine biosurfactants, structural diversity, glycolipids, lipopeptides, oil degradation, biotechnological application, biosynthetic mechanisms, heterologous expression, synthetic biology

## Abstract

Biosurfactants are amphiphilic secondary metabolites produced by microorganisms. Marine bacteria have recently emerged as a rich source for these natural products which exhibit surface-active properties, making them useful for diverse applications such as detergents, wetting and foaming agents, solubilisers, emulsifiers and dispersants. Although precise structural data are often lacking, the already available information deduced from biochemical analyses and genome sequences of marine microbes indicates a high structural diversity including a broad spectrum of fatty acid derivatives, lipoamino acids, lipopeptides and glycolipids. This review aims to summarise biosyntheses and structures with an emphasis on low molecular weight biosurfactants produced by marine microorganisms and describes various biotechnological applications with special emphasis on their role in the bioremediation of oil-contaminated environments. Furthermore, novel exploitation strategies are suggested in an attempt to extend the existing biosurfactant portfolio.

## 1. Introduction

Microorganisms produce a large variety of secondary metabolites that are of interest for the biotech and pharma industries. A prominent example comprises biosurfactants, a very diverse group of lipids which have a polar, amphiphilic character in common containing both hydrophilic and hydrophobic domains within one molecule. As a consequence of the amphiphilic structure, these compounds lower interfacial tension allows, for instance, solubilisation of hydrophobic substances in water [1]. In nature, the biosynthesis of amphiphilic substances could open ecological niches for the production of microorganisms in different habitats, for example, by exploitation of hydrophobic substrates, enabling motility, or avoiding competitors [2,3]. 

More than 2000 distinct biosurfactant structures are currently known, covering chemically different families of compounds, but also groups of congeners, that is, structurally closely related compounds with minor structural variations [4]. This structural diversity of biosurfactants implies a large variety of biological and physicochemical properties. Frequently highlighted properties of biosurfactants include low critical micelle concentrations (CMC), strong surface tension reduction, metal ion complexation, prominent bioactivities, and low eco-toxicity. A low CMC implies that such biosurfactants exert their function at much lower concentrations than many chemically produced surfactants [5]. Bioactivities of biosurfactants include antibacterial, antifungal and anti-tumour effects. At the same time, ecological aspects are considered as important, as biosurfactants can be produced from renewable resources and they exhibit low eco-toxicity in connection with supreme biological degradability preventing environmental accumulation [6,7]. 

These properties form the basis of the pronounced interest in this class of metabolites [8] for biotechnological applications, for example, as detergents, wetting agents for hydrophobic surfaces or fibres, and emulsifiers. In addition, they are used in food, cosmetics, and as pharmaceuticals [8]. To date, research regarding the technological access to biosurfactants has mostly focused on soil-isolated microbes, predominately species of *Bacillus, Pseudomonas*, or yeasts. More recently, however, marine habitats are considered as a prolific source for the discovery of microorganisms with a specialized metabolism and physiology that produces a variety of useful compounds such as biosurfactants [9,10,11,12]. This article aims to provide an overview of the structural diversity of low molecular-weight biosurfactants produced by marine microbes reported until May 2019. Furthermore, applications for the degradation of marine oil spills are expounded. Finally, strategies for future biotechnological exploitations of such compounds are discussed.

## 2. Structural Diversity of Biosurfactants 

Biosurfactants produced by different microorganisms exhibit an immense spectrum of diverse chemical structures. The amphiphilic character of biosurfactants is typically formed by both hydrophobic and hydrophilic components with the hydrophobic part usually comprising saturated or unsaturated fatty acids, hydroxy fatty acids, or fatty alcohols with a chain length between 8 and 18 carbon atoms. The hydrophilic components are constituted either by small hydroxyl, phosphate or carboxyl groups, or by carbohydrate (such as mono-, oligo-, or polysaccharides) or (poly-)peptide moieties. Biosurfactants are predominantly anionic and non-ionic compounds. It has been assumed that cationic biosurfactants exhibit higher toxicity and are thus found only rarely [4]. 

Besides the grouping by charge, surface-active secondary metabolites can be broadly classified into high and low molecular weight biosurfactants [13,14]. The structural diversity of the latter group can be further subdivided into fatty acids, lipoamino acids, lipopeptides, and glycolipids as outlined in more detail in the following section.

### 2.1. High Molecular Weight Polymeric Biosurfactants/Bioemulsifiers

High molecular weight polymeric (HMW) biosurfactants, suggested to be referred to as bioemulsifiers to distinguish them from low molecular weight metabolites [15], are produced by many bacteria of different species. They constitute polysaccharides, proteins, lipopolysaccharides, lipoproteins, or complex mixtures of these compounds referred to as lipoheteropolysaccharides. The prototypical HMW bioemulsifier emulsan is produced, for instance, by *Acinetobacter calcoaceticus* RAG-1, an isolate from the Mediterranean Sea [16,17]. It consists of a heteropolysaccharide backbone with a repeating tri-saccharide unit. This repeating unit probably consists of *N-*acetyl-d-galactosamine, *N-*acetylgalactosamine uronic acid and a di-amino-6-deoxy-d-glucose [4,18] with fatty acids (FA) covalently linked to the polysaccharide through ester linkages. In spite of the structural complexity, the genes encoding emulsan synthesis by *A. calcoaceticus* RAG-1 were identified and shown to be organised in a single *wee* gene cluster of 27 kilo base pairs [19]. 

Amphiphilic proteins may also be regarded as polymeric surfactants. They constitute a remarkable class of little-explored and biotechnologically hardly targeted biosurfactants. Naturally occurring foams often contain proteins with foam-stabilising properties. A particularly interesting example from the non-microbial world is ranaspumine produced by tropical frogs that use foam nests to protect their eggs. Another likewise foaming protein is latherin, mainly known from horse sweat [20]. Hydrophobins are small proteins secreted by different filamentous fungi [21] which are also surface-active and may represent promising targets for biotechnology [22].

### 2.2. Low Molecular Weight Biosurfactants

Low molecular weight (LMW) biosurfactants range from simple free fatty acids and phospholipids to amino acids linked to lipids, to lipopeptides, and glycolipids.

**Fatty acid and phospholipid** derivatives may act as surface-active substances. Strong reduction of surface and interfacial tensions were, for instance, observed for branched fatty acids known as corynomycolic acids with chain lengths of C_12_–C_14_ [23,24,25].

(Hydroxy) fatty acids bound to proteinogenic or non-proteinogenic amino acids form **lipoamino acid** biosurfactants, for example, ornithine lipids, lysine lipids, *N*-acyltyrosines or cerilipin containing ornithine and taurine produced, for example, by *Myroides* sp., *Gluconobacter cerinus* and *Nitrosomonas europaea* [26,27,28,29].

**Lipopeptides** are a very prominent group of LMW biosurfactants derived from amino acids. These often cyclic depsipeptides are produced by various clades of microorganisms including the bacterial genera *Bacillus*, *Lactobacillus*, *Streptomyces*, *Pseudomonas* and *Serratia.* As a result of their non-ribosomal origin, they often contain non-proteinogenic amino acids such as d-enantiomers. Many lipopeptides show not only a reduction of surface tension, but also significant bioactivities [30,31], with the antibiotics daptomycin from *Streptomyces roseosporus* and polymyxin B from *Paenibacillus polymyxa* as prominent examples [32,33]. Surfactin produced by different species of the genus *Bacillus* is considered as one of the most effective biosurfactants of all, stated to reduce the air/water surface tension from 72.5 mN/m down to 27 mN/m [34,35].

**Glycolipids**, similar to the emulsan repeating unit, consist of mono- or oligosaccharides and a lipid moiety. Typical sugars forming the hydrophilic part are glucose, mannose, galactose, glucuronic acid, or rhamnose, while the hydrophobic part consists of saturated or unsaturated fatty acids, hydroxy fatty acids or fatty alcohols. The best-explored groups comprise sophorolipids, mannosylerythritol lipids, trehalose lipids and rhamnolipids [4].

Sophorolipids contain the di-saccharide sophorose and, predominantly, 17-hydroxyoleic acids. They usually form lactones, but also occur in the acidic form, that is, without ring closure [36]. The yeast *Starmerella bombicola* is the most prominent producer of sophorolipid [37]. Mannosylerythritol lipids (MELs) are also mainly known from fungal species, such as *Pseudozyma* sp. and *Ustilago maydis.* MELs comprise 4-*O*-β-d-mannopyranosyl-d-erythritol in their carbohydrate moiety, which displays diverse acylation patterns and a variety of chain lengths of the acyl groups [38,39]. Trehalose lipids contain the di-saccharide trehalose, which is acylated with long-chained, straight or α-branched 3-hydroxy fatty acids called mycolic acids and are mainly known from Actinobacteria like *Mycobacterium*, *Arthrobacter* and *Rhodococcus* species [40,41]. Rhamnolipids are best known from the pathogenic bacterium *Pseudomonas aeruginosa* but are also reported from different *Burkholderia* spp. [42]. They comprise one or two α-l-rhamnose units, commonly coupled to two 3-hydroxy fatty acid moieties via a glycosidic linkage that defines different congeners with a chain length between 8 and 16 carbon atoms [42,43]. The length of acyl chains within a certain range appears to be species-specific to some extent [44]. Moreover, decorations like methylations and acetylations are reported occasionally [42]. Further reported glycolipids include glucose lipids, cellobiose lipids, polyketide glycosides, isoprenoid and carotenoid glycolipids [45].

The phylogeny of biosurfactant-producing microorganisms is as diverse as the chemical composition of these metabolites. Beyond the already mentioned examples, known biosurfactant producers are, for instance, Firmicutes like *Lactococcus* sp., Proteobacteria like different Pseudomonads or *Serratia* spp., Actinobacteria like *Streptomyces* spp., and fungi like *Cryptococcus* sp. [46]. Producer strains were isolated from many different habitats, i.e. fresh water, soil, pathogen biofilms, leaf surfaces and industrial effluents, oil wells and marine environments [47,48].

## 3. LMW Biosurfactants from Marine Sources

**Figure 1:**
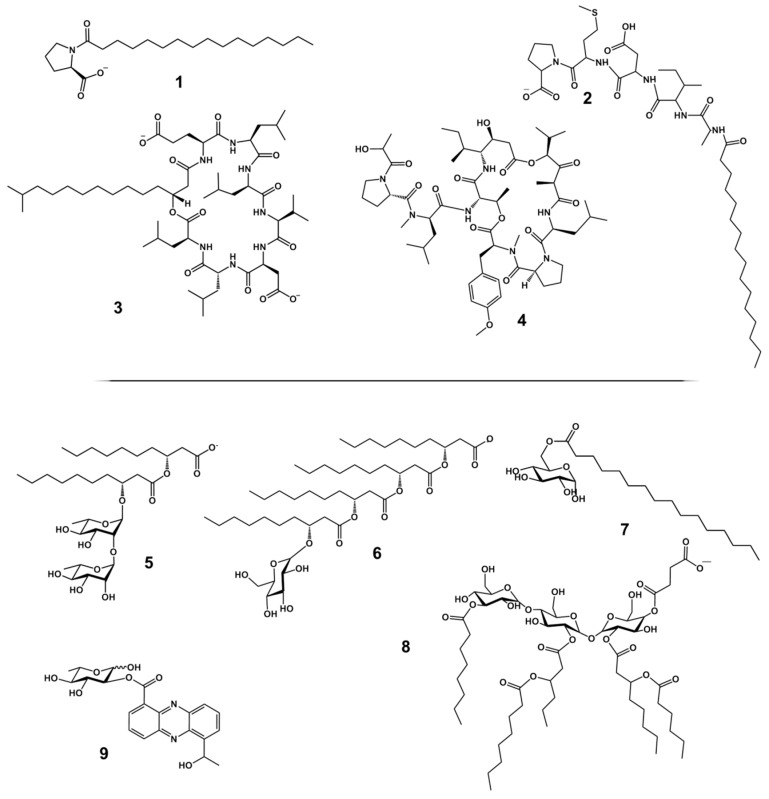
Structural diversity of marine low molecular weight (LMW) biosurfactants. **1**–**4** representative lipoamino acid and lipopeptide biosurfactants: **1** proline lipid (*Alcanivorax dieselolei*); **2** rhodofactin (*Rhodococcus* sp.); **3** surfactin (*Bacillus subtilis*); **4** didemnin B (*Tristrella* sp.). **5**–**8** representative glycolipid biosurfactants: **5** di-rhamnolipid (*Pseudomonas aeruginosa*); **6** glucose lipid (*Alcanivorax borkumensis*); **7** glucosyl palmitate (*Serratia marcescens*); **8** tri-glucose-tetraester (*Rhodococcus* sp.); **9** 2-l-quinovose - phenazine ester (*Streptomyces* sp.).

Several decades ago, marine habitats had already attracted attention as a prolific repository harbouring surface-active molecules [49,50,51,52] and different aspects regarding these molecules have since been reviewed including the occurrence and isolation of producers [53], pharmacological activities [54] or other particular applications [53,55,56,57]. The production and potential applications of HMW bioemulsifiers have been reviewed elsewhere [12,58]. The focus of this report is on LMW biosurfactants. The following section provides an overview of the molecular structures (selected examples in Figure 1), the respective microbial producers, and their marine habitats (Table 1).

**Fatty acids** may be seen as the simplest amphiphilic metabolites. The surface-activity of two marine microbes was attributed to secreted fatty acids: The marine yeast *Aureobasidium pullulans* YTP6-14 was shown to produce massoia lactones as surface-active compounds, which are lactonised hydroxy fatty acids [59], usable as a fragrance. *Cobetia* sp., a Gammaproteobacterium, secretes linear 3-hydroxy fatty acids [60]; hydroxy fatty acid dimers designated as rubiwettin R1 were isolated from cultures of *Serratia rubidaea* [61], which is identical to the marine bacterium described in 1944 as *Serratia marinorubra*.

**Lipoamino acid** production was also reported for a few marine bacteria. The flavobacterium *Myroides* sp. was found to produce a crude oil emulsifying metabolite consisting of the non-proteinogenic amino acid ornithine linked to a 3-hydroxy-13-methyltetradecanoic acid diester [62]. Furthermore, production of nearly identical proline lipids with long chain fatty acids (**1**) was described for two bacteria of remarkable evolutionary distance, namely the Gammaproteobacterium *Alcanivorax dieselolei* and the Actinobacterium *Brevibacterium luteolum* [63,64].

Dominant phyla among the producers of **lipopeptides** are Actinobacteria [64,65,66,67] and Firmicutes like *Bacillus* sp. [69,70,71,72,73,74,75,76,77,78,79,80,81,82,83,84]. Actinobacterial compounds include rhodofactin produced by *Rhodococcus* sp. (**2**) and a notable lipopeptide of *N. alba* that was recently stated to contain a phenylalanine dipeptide [65,68], and hence appears structurally special among the multitude of linear and cyclic lipo-oligopeptides.

Many of the lipopeptides described to be isolated from marine Firmicutes do not occur exclusively in marine strains: They were initially isolated from non-marine strains, prior to the identification of their production in marine relatives. This pertains, in particular, to surfactins (**3**), lichenysin, fengycin, iturins, pumilacidin, plipastatin, polymyxin B, fusaricidin and bacillomycin F. However, the respective studies unveiled novel producers of these surfactants [69,70,71,72,73,74,75,76,77,78,79,80,81], which may be useful, for example, for environmental applications such as surfactant-producing inoculum (see Section 6) because of their adaptation to high salinities or low temperatures. The known portfolio of the Firmicutes’ lipopeptides was expanded by the discovery of completely new compounds produced by marine isolates, often associated with distinct bioactivities. For example, tauramamide and ester derivatives thereof as well as aneurinifactin [82,83] have been introduced first as antibiotics. Didemnin B (**4**) was first described as an anti-tumour agent [107], but recently reported to be the surface-active agent of a *Bacillus amyloliquefaciens* strain isolated from the Red Sea after enrichment of cultures on crude oil. Prior to this publication, didemnin B was commonly considered to be produced by the alphaproteobacterial genus *Tistrella* isolated from geographically widely distributed sites (Caribbean, Pacific, Red Sea) [85].

Beyond *Tistrella* spp., there are only a few further reports on marine proteobacterial producers of lipopeptides: *Achromobacter* sp. HZ-01, a hydrocarbon-degrading Betaproteobacterium [86,108], and epiphytic marine Pseudomonads that were shown to produce a group of antibacterial viscosin-like lipopeptides designated as massetolide A-H [87]. The serine-rich lipopeptide pontifactin was likewise described as an antibiotic; it is produced by the Flavobacterium *Pontibacter korlensis*, which was isolated from petroleum-contaminated seawater [89]. 

Non-ribosomal peptides, like lipopeptides, along with polyketides and hybrids of both, have typically been considered primarily for their often-prominent bioactivities. Marine organisms are regarded as a promising source for such natural products [109]. The later appearance of pharmacologically active compounds like polymyxin B and didemnin B in screenings for surface-activity exemplifies that further lipopeptide biosurfactants may be already explored on a structural level, but not assessed as such until today. Hence, more marine lipopeptide biosurfactants may already be known, but have been investigated thus far for their bioactivity only and have not been evaluated for their surface-activity.

**Glycolipids** are well-known for relatively easy large-scale production and various applications as surfactants; in particular, the yeast-derived sophorolipids, MELs, and rhamnolipids (**5**) from *P. aeruginosa* (**5**). This bacterium is known to be ubiquitously distributed; hence, it is not surprising that it was also isolated from marine samples as a producer of rhamnolipids [90,91,92]. However, *P. aeruginosa* is also a well-known human pathogen, making it less attractive for industrial applications. Recently, a non-pathogenic producer of *P. aeruginosa*-like rhamnolipids was described with the marine *Pseudomonas* sp. MCTG214(3b1), a potential future alternative for industrial production [94]. Notably, one of the marine *Pseudomonas* isolates apparently secretes solely mono-rhamnolipids [93], an uncommon feature among wild type rhamnolipid producers, which are to the largest extent capable of the synthesis of mixtures of mono- and di-rhamnolipids [110]. As a result of such reports, the marine environment has been considered as a promising source for novel rhamnolipid producer strains [111].

Structurally related to rhamnolipids are the glucose lipids produced by *S. rubidaea* (rubiwettin RG2 [61]) and *Alcanivorax borkumensis.* The latter is an obligate alkane-degrading Gammaproteobacterium, which is globally distributed in marine habitats and was once isolated from the North Sea as strain MM1, later defined as *Alcaligenes*, before it was finally classified as *A. borkumensis* [49,98]. This strain produces a glucose lipid glycosidically-linked to four 3-hydroxy fatty acids [51] (**6**); the terminal acid group can be linked to glycine [97]. Interestingly, the glycine-containing species were found associated with the bacterial envelope, whereas only the glycine-free species were recovered from culture supernatants, suggesting that the free glycolipid is released from the cell by cleavage of the terminal amide linkage of the membrane-stored lipid. However, the respective enzyme, like all other enzymes involved in the biosynthesis, remains unknown. The free glucose lipids of *A. borkumensis* were shown to exhibit remarkably low ecotoxicity in comparison to synthetic surfactants and even to other glycolipid biosurfactants [6,51].

Ester linkages, instead of glycosidic bonds, were reported for glucose lipids from a mangrove-isolated *Buttiauxella* sp. and a coral isolate identified as *Serratia marcescens.* Here, glucose moieties are esterified with middle and long chain length fatty acids (C14 to C18), respectively, to form, for example, glycosyl palmitate (**7**) [95,96]. For proteobacterial glycolipids, ester-linked fatty acids are very uncommon. However, they represent a common feature of actinobacterial glycolipids [42] where esters occur consisting of fatty acids, succinate or mycolic acids and trehalose or tri-glucose units (**8**). Other marine actinobacteria are reported to produce extraordinary rhamnolipids with a single subterminal additionally hydroxylated 3-OH fatty acid [102].

Among Actinobacteria, *Streptomyces* spp. are famous for the production of most diverse natural products including important antibiotics. In line with that, the production of different biosurfactants is also reported for various representatives of this genus [42]. However, marine Streptomycetes seem to be underexplored regarding biosurfactants production, as only two publications report on the production of di-rhamnolipids [103], and different l-quinovose phenazine esters containing a phenazine instead of lipids as hydrophobic moiety [42,104] (**9**). Phenazines are heteroaromatic compounds produced by many Pseudomonads and Streptomycetes [112], but this is the only report of a phenazine biosurfactant. Firmicutes are very rarely described as glycolipid producers. The only marine example is, to the best of our knowledge, a biofilm inhibiting threose-di-lipid from a snail epiphytic *Staphylococcus lentus* [105].

The number of reports describing biosurfactant producers isolated from marine sources does by no means match the number of solved molecular structures. Hence, many new structures of surface-active molecules from the sea need to be determined to extend the portfolio of marine biosurfactants.

## 4. Biosynthetic Mechanisms

The lack of molecular structures coincides with the missing knowledge of the underlying biosynthetic pathways leading to the production of many marine biosurfactants. Most of the known pathways were explored in studies using non-marine strains producing compounds that also appeared in marine samples, whereas biosynthesis pathways of surfactants exclusively occurring in marine phyla are barely known (Table 2). The following section describes the biosynthetic principles for the production of different biosurfactant classes based on known pathways.

**Figure 2:**
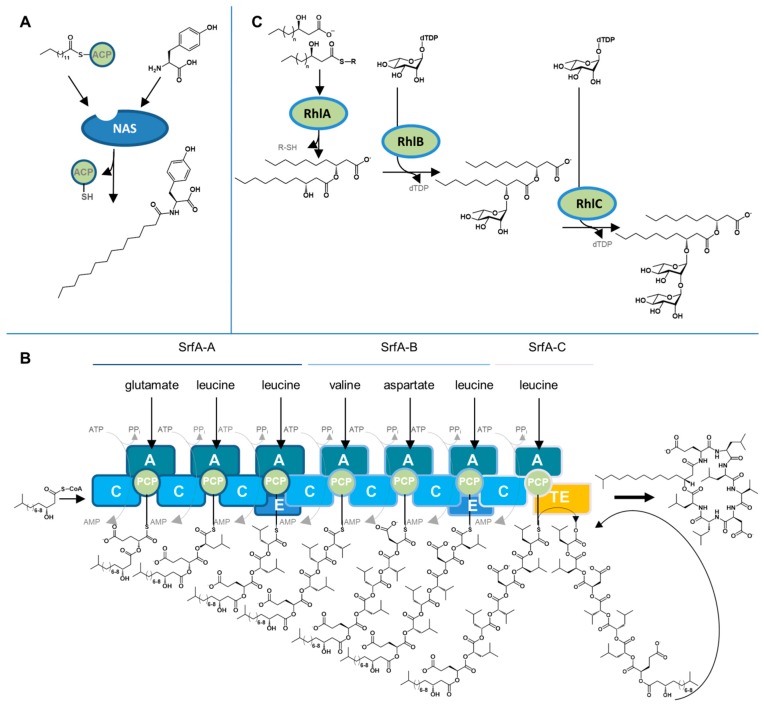
Biosynthetic pathways leading to low molecular weight biosurfactants. (**A**) Synthesis of lipoamino acid *N*-myristoyltyrosine *via N*-acyl amino acid synthase (NAS). ACP, acyl carrier protein. (**B**) Lipopeptide surfactin biosynthesis by the non-ribosomal peptide synthetases SrfA-A, SrfA-B, and SrfA-C, showing the principle of the modular non-ribosomal peptide biosynthesis. C, condensation domain; A, adenylation domain; PCP, peptidyl carrier domain; E, epimerisation domain; TE, thioesterase domain. (**C**) Biosynthesis of 3-(3-hydroxyalkanoyloxy)alkanoic acid, mono-rhamnolipids and di-rhamnolipids, constituting fatty acids and glycolipid biosurfactants, respectively, by the enzymes RhlA-C of *P. aeruginosa*.

Fatty acids and hydroxy fatty acids presumably originate from the primary carbon metabolism. The biosynthesis of lipoamino acid biosurfactants from marine bacteria was not described. It might resemble the biosynthesis by environmental bacteria of *N*-acyl amino acids (Figure 2A) from activated fatty acids and amino acids catalysed by acyltransferases of the *N*-acyl amino acid synthase class [122,123,124].

Lipopeptide producing microbes have gained particular interest for their potential applications as biocontrol strains or as producers of pharmaceutically active compounds [33,125]. In contrast to the polypeptide chains in proteins, lipopeptides like surfactin (**3**) are not formed ribosomally by translation of an mRNA template, but by special, non-ribosomal peptide synthetases (NRPS), which are built up in a modular fashion (Figure 2B). Each module leads to the addition of one amino acid to the peptide chain; furthermore, the NRPS can catalyse reactions including the incorporation of lipids, epimerisation or lactonisation [126,127]. A prototypic module consists of three domains: A condensation, an adenylation, and a peptidyl carrier domain. Here, the adenylation domain is responsible for the amino acid selectivity of the respective module. Additional epimerisation domains are commonly responsible for the transformation of l– to d-amino acids. Hence, the composition of non-ribosomal peptides is not limited to the proteinogenic amino acids.

The surfactin biosynthetic molecular machinery (Figure 2B) constitutes one of the best studied NRPS. It involves an NRPS consisting of three multifunctional proteins encoded in the *srfA* operon, which comprises *srfA-A*, *srfA-B* and *srfA-C*. The proteins SrfA-A and SrfA-B consist of three modules each, SrfA-C contains one module including a thioesterase domain. The condensation domain of the first module recognises a 3-hydroxy fatty acid starter molecule, typically 3-hydroxy-13-methyl-myristic acid, to which seven amino acids (l-Glu, l-Leu, d-Leu, l-Val, l-Asp, d-Leu and l-Leu) are sequentially added by seven modules. Product release and lactonisation of the depsipeptide are finally catalysed by the thioesterase domain of the termination module SrfA-C [113,114]. Analogous biosynthetic machineries were described for the synthesis of lichenysin [115], fengycin [117], iturin [118], plipastatin [119], polymyxin B [120], fusaricidin [121,128], and massetolide/viscosins [88]. The didemnin B biosynthesis by *Tistrella* sp. was also described in detail including an unusual post-synthetase activation mechanism [85].

The biosynthesis of rhamnolipids by *P. aeruginosa* (Figure 2C) probably constitutes the best-known example of a biosynthetic pathway for a bacterial glycolipid which is reported for non-marine and marine strains. The formation of mono- and di-rhamnolipids (**5**) is catalysed by two different glycosyltransferase units, that is, rhamnosyltransferase I and II. The products of genes *rhlA* and *rhlB*, which are organised as a bicistronic operon, were originally designated rhamnosyltransferase I; however, recent studies proved the independent activity of both RhlA and RhlB proteins [129]. RhlA synthesises 3-(3-hydroxyalkanoyloxy)alkanoic acids (HAA) from activated hydroxy fatty acids, while the glycosyltransferase RhlB catalyses the condensation between the dTDP-l-rhamnose and the HAA to form mono-rhamnolipids. Notably, HAA themselves are already surface-active metabolites, which are released to the cell’s environment as biosurfactants [130]. The gene *rhlC*, which is localised at another chromosomal site apart from *rhlAB* in *P. aeruginosa* (but interestingly not in *Burkholderia* spp.), encodes for rhamnosyltransferase II. This protein catalyses the production of di-rhamnolipid (l-rhamnose-l-rhamnose-3-(3-hydroxyalkanoyloxy)alkanoic acid) from mono-rhamnolipid and dTDP-l-rhamnose [110].

As far as we know, the biosynthesis of other marine glycolipids has not been elucidated yet. Proteins like the RhlA homolog AML58231 of *S. rubidaea* strain 1122 (showing 43% identity to RhlA of *Burkholderia glumae* at a query coverage of 94%) may be involved in the synthesis of rubiwettin.

Apparently, the biosynthesis of the majority of biosurfactants is strictly regulated. As a consequence, they are only formed under distinct conditions, for instance, dependent on the cell density, limiting growth conditions or the presence of hydrophobic nutrients [4,47,48,131,132]. Presently, most of the known biosynthetic pathways for marine biosurfactants originate from studies of related non-marine bacteria. The availability of inexpensive sequencing methods and bioinformatic analysis tools now allows deducing putative biosurfactant biosynthetic genes from genome sequence data as discussed, for example, for *Alcanivorax* glucose lipids [133] or *Rhodococcus* trehalose lipids [134].

## 5. Prospecting for Novel Biosurfactants

The marine environment comprises many different habitats, including those that appear challenging for life because of extreme temperature, high pressure and salinity, poor nutrition or toxic pollutants. Nonetheless, almost every niche inhabits specifically adapted microorganisms able to cope with these challenges by using designated tools to survive, which were developed by evolution over millions of years. Biosurfactant production may constitute such a tool which may, for example, enable adaption to cold environments (present in many marine habitats), but the underlying mechanisms are unknown [57,135]. The studies that were taken as a basis of the review do not indicate biosurfactant production as a feature predominantly of microbes from cold habitats. In fact, samples were derived from locations in all climatic zones.

The studies published so far suggest that biosurfactant producers may be identified within marine samples mainly in communities associated with invertebrates and in oil-polluted environments. The flora associated with filter-feeding marine invertebrates like sponges and tunicates is known to be an astonishing source for novel complex and bioactive secondary metabolites in general [136,137,138], and likewise constitutes a promising source for surface-active secondary metabolites. In oil-polluted environments, biosurfactants/bioemulsifiers may help to utilise the water-insoluble polluting chemicals as a nutrient source [139], as outlined below. This section will discuss the role of biosurfactants in the natural degradation of hydrocarbon spills.

Crude oil is a complex mixture of different aliphatic and aromatic hydrocarbons with generally low bioavailability [140], that is, the hydrophobic character of crude oil prohibits the uptake and metabolism by most microbes. Therefore, crude oil is extremely persistent in the environment, causing a variety of harmful ecological effects. Hydrocarbonoclastic bacteria, in contrast, are not only able to proliferate in environments polluted with crude oil but are even able to “make a meal of oil” [140], using the crude-oil as a preferred carbon source by solubilising and oxidising the alkanes [141]. A large part of the inhabitants of such polluted sites is considered to represent highly specialised organisms perfectly adapted to this niche [142]. Prominent examples for such obligate oil-degrading marine bacteria, hydrocarbonoclastics *sensu strictu*, are representatives of the genera *Alcanivorax*, *Cycloclasticus*, *Marinobacter*, *Oleispira* or *Thalassolituus*, which can constitute up to 90% of the microbial population during an oil spill [140,143,144,145]. Besides, more generalistic bacteria from the genera *Pseudomonas* [90], *Paracoccus* [99] or *Rhodococcus* [101] have been isolated from crude oil-contaminated marine samples and showed the ability to degrade oil/alkanes.

The ability of hydrocarbon degradation often comes along with the production of biosurfactants; thus, it is presumed that most hydrocarbonoclastic bacteria are able to produce at least one biosurfactant [46,141]. Within the *Alcanivorax* genus, *A. borkumensis* synthesises glucose-HAA **(6)**, whereas *A. dieselolei* is known for the production of proline lipids (**1**) and *A. hongdengensis* is described to produce a lipopeptide of currently unknown structure (Table 1, [146]). The development of apparently unrelated biosynthetic pathways for structurally distinct biosurfactants within a single genus may also indicate an underlying evolutionary pressure towards surfactant production associated with this ecological niche. However, there are still many hydrocarbonoclastic species without reported ability to produce surface-active compounds. Reasons may include that the respective compounds are not yet discovered, the bacteria rely as secondary consumers on surfactants produced by others [140] or they use alternative strategies to facilitate access to alkanes, for example, the formation of outer membrane vesicles [147].

The biological benefits of surfactant production for hydrocarbonoclastic bacteria include an increase in bioavailability of hydrophobic carbon sources, that is, the rate of substrate mass transfer into the cell [148] and improved efficiency of cell-adherence to the hydrophobic alkane phase [149,150,151]. It remains to be demonstrated whether one of these mechanisms is dominant or if both interact, at least to some extent [46,152,153]. For instance, for *A. borkumensis* it was assumed that the two glycolipid variants produced by this bacterium are involved in differential mechanisms to facilitate hydrocarbon uptake. The function of the cell-associated glycine-containing biosurfactant, although its production is apparently not dependent on the supplementation of alkanes, was assumed to increase of the cell’s hydrophobicity. The altered cell surface hydrophobicity is the prerequisite for attachment and biofilm formation on hydrocarbon droplets which is typical for this bacterium [151,154]. The extracellular glucose lipid, in contrast, should promote the creation of micelles with water-insoluble fractions [155].

The frequent occurrence of surface-active molecules among the often highly specialised alkane-degrading microorganisms renders them exceptionally interesting for biotechnological applications, for example, with regard to the fast bioremediation of oil spills. In addition, novel compounds can be identified and used for different applications by straightforward enrichment, isolation and identification [53,60,66,83].

## 6. Applications of Biosurfactants

The physical and biological properties of biosurfactants render them usable in a variety of different fields as detergents, wetting and foaming agents, solubilisers, emulsifiers and dispersants [155,156]. In addition, the chemical industries increasingly recognise the importance of these bio-based compounds as drivers towards a bio-based economy [157]. This industrial interest is indicated by an increasing number of patents describing various utilisations of these compounds [110,158]. Furthermore, numerous studies have been conducted to explore further fields of application for biosurfactants as outlined below.

### 6.1. Medical Applications

Many surface-active lipids, especially lipopeptides, have been described as having bioactivity. Antimicrobial activities of such biosurfactants have been described in several recent studies and reviews [159,160,161,162,163,164,165], often with particular emphasis on marine compounds [54,83,89]. Interestingly, some biosurfactants appear to consist of mixtures of molecules exhibiting different modes of action. As an example, within the naturally occurring rhamnolipids mixture, it was suggested that mono-rhamnolipids exhibit a bacteriostatic effect, while di-rhamnolipids were found to act bacteriocidal on *P. aeruginosa* [166]. In some cases, biosurfactants were shown to exert reinforcing or even synergistic effects with antibiotics, for example, by enhancing the penetration efficiency of the antibiotic into the cell [167,168,169,170].

Moreover, biosurfactants often prove especially effective in the **disruption of biofilms** [89,96,105,166,171], which protect pathogenic bacteria from immune cells and antibiotics. Biofilm disruption may, therefore, support the application of antibiotics. Although biosurfactants are often secreted by pathogenic organisms as an important factor in the development of biofilms in the first place, they are also able to disrupt them. Both effects may be related to the formation and maintenance of water channels within the biofilms. These are essential for nutrient supply; however, further extension of such channels by additional biosurfactants leads to the dissociation of parts of the biofilm [171]. Interference with biofilm regulating quorum sensing systems has been reported [60]. Furthermore, some lipopeptides, in particular, polymyxins, are applied to adsorb lipopolysaccharide endotoxins and may, thus, contribute to relieve inflammation or avoid sepsis reactions [172]. 

Some biosurfactants have furthermore received a lot of attention because of their **anti-tumour cell activity** [53,165]. Although some surface-active natural products have successfully entered clinical trials like the marine compound didemnin B, reports of in vitro anticancer activities of biosurfactants should be treated with some caution, as shown for sophorolipids. Here, translation to in vivo systems resulted in even opposite effects, i.e. an enhanced metastasis by the release of cells from the primary tumour [173]. In addition to naturally occurring molecules, modification approaches using mutasynthesis, semisynthesis or molecular engineering of the production machinery (see Section 7) have been described, specifically for lipopeptides aiming to change or improve bioactivities [174,175,176].

Furthermore, biosurfactants have been successfully tested for the **treatment of burn wounds**, where they facilitated wound healing and reduced collagen content-related scar-formation [177].

### 6.2. Food

The usage of non-toxic and biodegradable ingredients is crucial for **food production**. In view of the fact that the continuing growth of the human population requires measures to increase agricultural productivity, biosurfactants can provide support by (i) enhancement of beneficial microbe-plant interactions, (ii) protection from phytopathogens, (iii) soil improvement, and (iv) stimulation of effective foliar fertilisers uptake [178,179,180,181]. In addition to their applications in food production, the antimicrobial and antibiofilm effects can also be put to use in the food industry to sanitise production equipment and prevent food spoilage [182]. Applications in food products themselves mainly serve consistency control, stable solubilisation of ingredients like flavour oils, and fat stability [183,184]. Furthermore, compounds like Massoia lactone, which exhibits a pleasant odour and flavour, can be obtained from marine fungi [59], which may be applied as a novel natural resource for the compound as an alternative to the currently dominant production route applying synthetic chemistry. Finally, wastes of the food industry may be used as feedstock to produce biosurfactants in the first place [183]. A smart combination of these applications may finally enable a “cradle to cradle approach” as required by a strictly circular bioeconomy.

### 6.3. Consumer Products

**Cosmetics-**related applications of biosurfactants are mainly based on their emulsifying, solubilising, wetting, foaming and dispersing properties, which support the solubilisation of hydrophobic ingredients in the products as well as their delivery through the skin barrier [185,186,187]. The low irritancy and high skin compatibility of many biosurfactants constitute a strong advantage over non-natural counterparts [188]. Biosurfactants with antimicrobial activity also see use in cosmetic products as recently reviewed [188,189,190,191].

Due to their intrinsic emulsifying and surface-active properties, application of biosurfactants as a nature-derived alternative to chemical detergents for **cleaning purposes** appears obvious [192,193,194]. In addition to their biodegradability, which makes them less threatening upon environmental release, the interest to include biosurfactants in such applications is driven by their frequently observed activity over a broad range of temperature, pH, and salinity [195,196]. Biosurfactants produced by cold-adapted microbial species common in marine habitats are of particular interest for less energy-demanding low-temperature applications [56,135]. Despite all the advantages, the establishment of biosurfactants as a sustainable alternative to inexpensive conventional surfactants is still impeded by the fact that the bulk detergent market is strongly driven by cost-effectiveness. Although biosurfactants currently appear too expensive to compete, few cleaning products containing glycolipids have been commercialised as niche products and successfully marketed emphasising sustainability and biodegradability.

### 6.4. Bioremediation

Due to their biodegradability and low ecotoxicity, biosurfactants appear predetermined for applications involving a release into the environment. Such processes are of great interest, since the accumulation of petroleum hydrocarbons, heavy metals and other pollutants constitute a growing global concern for terrestrial and marine environments [150,197,198,199]. Examples are **remediation processes** to treat polluted sites by applying bioremediation concepts that rely on bio-based compounds. In line with their natural function, biosurfactants can be applied to promote water solubility of hydrophobic pollutants, which facilitates their removal [110,200,201]. Here, they appear as an ecologically supreme alternative to classical surfactants applied in remediation [202,203]. As enhanced solubility or emulsification also facilitates the cellular uptake of contaminants, biosurfactants are especially useful in remediation techniques combining biochemical supplements and living organisms. 

### 6.5. Enhanced Recovery of Fossil Resources

Despite known drawbacks, like the danger of environmental pollution, crude oil and petroleum-based products still play a significant role in modern society. However, common oil extraction procedures are only capable of recovering 10%–40% of the content of oil reservoirs [204], calling for new concepts to enhance oil recovery. The microbially enhanced oil recovery (MEOR) as one of these concepts utilises the natural capabilities of certain microorganisms to solubilise oil, for example, by the help of biosurfactants (Section 5). It includes (i) stimulation of the growth of reservoir-indigenous hydrocarbonoclastic bacteria, for example, by offering additional nitrogen sources; (ii) injection of selected consortia of such bacteria into the reservoir; or (iii) *ex situ* production and addition to reservoirs of microbial compounds including biosurfactants [205]. Accordingly, several biosurfactants have been proven to enhance oil recovery [206].

Gas hydrates, which are ice formations with trapped molecules of gas (typically methane) inside, represent another repository of stored energy and carbon, mainly found beneath the sea floor and in permafrost regions. These repositories are regarded as a next-generation energy source [207] and, therefore, current efforts aim to develop technologies for the efficient and safe storage and transportation of gas hydrates. Studies showed that biosurfactants are capable to promote methane hydrate re-formation and thus to improve the storage [208,209].

### 6.6. Industrial Processes

Biosurfactants are discussed as facilitators of several industrial processes including cooling and biorefinery. Industrial process **cooling**, cold-storage, and air conditioning systems may rely on ice slurry, a homogenous mixture of water and small ice particles. However, the particle size is detrimental to flow and equipment. Di-acetylated MELs were shown to prevent agglomeration in ice-water slurries by stabilising small ice particles and thus preventing the formation of bigger crystals [210]. MEL additives were furthermore shown to improve flow properties of biodiesel and hence improve its performance at low temperature [211]. Biosurfactants are also applicable to improve the degradation of complex biomass in so-called **biorefinery processes** to exploit alternative industrial resources. Here, their supplementation may enhance enzymatic lignocellulose degradation efficiency, probably by the improvement of substrate binding of the applied cellulases [212]. Another study reported increased hydrogen production from organic solid waste through surfactin and saponin supplementation [213].

In summary, biosurfactants are useful for a variety of highly diverse applications. However, with the exception of studies concerning medicinal application, most reports currently focus on a small set of well-characterised compounds, that is, sophorolipids, rhamnolipids, mannosylerythritol lipids and, from the lipopeptide group, surfactin and related molecules. Other biosurfactants will surely open further opportunities or perform even better in already discussed applications. In particular, biosurfactants from marine environments may be of interest because they may be adapted to work efficiently in cold or saline environments and therefore open up further fields of application [57]. Most often, temperature or salinity of a habitat is not stated in reports on novel biosurfactant producing isolates. Hence, the physicochemical properties of those products cannot be necessarily deduced from the source and have to be characterized in vitro. Current difficulties to supply industries with sufficient amounts of novel compounds [214] require the development of appropriate production strategies and processes. The structural elucidation and physicochemical characterisation of such novel biosurfactants will also lead to novel applications.

## 7. Perspectives for the Biotechnological Exploitation of Marine Biosurfactants

The biotechnological production of non-marine biosurfactants is described in a multitude of studies in both, natural microbial producer strains and heterologous systems [37,110,215,216]. However, production strategies for compounds from marine producer organisms, predominantly bacterial species (see Section 3), have been explored only occasionally. Examples include a study on *A. borkumensis*-derived glycolipids [52] and, very recently, another on *Rhodococcus*-derived trehalose lipids [217]. In the following, general aspects regarding future biotechnological exploitations of marine biosurfactants are discussed.

The production of biosurfactants by cultivating marine bacteria may pose technical challenges in establishing robust and feasible fermentation conditions [53]. Furthermore, intrinsic regulatory circuits may require the addition of oil for induction of the surfactant biosynthesis [218] (Table 1), which in turn makes subsequent downstream processing more complex. Therefore, recombinant production appears to be a more promising approach for accessing marine biosurfactants if sufficient knowledge about biosynthesis and genetics is available. Here, functional metagenomics [28,29], sequence data generated by next-generation sequencing of single producer strains or environmental DNA and a detailed understanding of the underlying pathways facilitated by steadily improved bioinformatic tools will allow the identification of a multitude of biosynthesis pathways leading to marine surfactants [219,220]. Heterologous biosurfactant production may even contribute to elucidating biosynthetic mechanisms as shown recently [129]. Currently, Pseudomonads, namely *P. aeruginosa and P. putida*, as well as *B. subtilis*, and *Escherichia coli*, are mainly used for the biotechnological production of surfactants of non-marine origin [110,215,216]. These strains can provide biosynthetic precursor molecules and exhibit sufficient tolerance towards the respective surface-active end products. Moreover, due to phylogenetic relationships, they feature genetic compatibility with a range of marine producer strains in terms of guanine-cytosine content and thus codon usage. However, several marine producer strains belong to actinobacterial clades, calling for the exploration of additional host systems for marine biosurfactant production. To fill this gap, Streptomycetes that represent valuable hosts especially for NRPS-derived compounds [176,221], may prove suitable as production hosts in future endeavours.

As a first step toward the implementation of recombinant biosynthesis in a chosen host, the biosynthetic genes need to be cloned and functionally expressed. Therefore, smart cloning methods represent a key enabling technology in natural product research in general, where a number of genes or large biosynthetic gene clusters need to be handled [222]. Besides conventional restriction-ligation cloning, diverse “smart” protocols are established including type II restriction enzyme-based cloning strategies [223,224] and restriction-independent methods like phage enzyme-mediated recombination in *E. coli* and in vitro homology-based methods [225,226,227]. In addition, yeast recombination has been increasingly used for successful cloning and engineering of biosynthetic genes, for example, encoding biosurfactant synthesis [228,229,230].

The transfer of the cloned genes into an expression host is usually carried out using well-established protocols including electroporation and conjugation methods [231,232,233,234]. The respective genes can be maintained on a plasmid or integrated into the bacterial chromosome, both being viable strategies. For recombinant biosurfactant production with *P. putida* and *E. coli*, plasmid-based gene expression has been used in most cases [130,235,236,237], whereas chromosomal integration of biosynthetic genes becomes increasingly popular in natural products research in general [222,238]. This also holds for *B. subtilis*, where chromosomal integration represents a common approach to confer gene expression for the production of biosurfactants and other natural products [120,230,239]. For future approaches, it should be considered that plasmid-based expression enables high flexibility, that is, the plasmid-encoded genes can easily be expressed in different strains with different metabolic backgrounds. However, the burden of plasmid replication can lead to certain instability of constructs [230,239,240]. Strategies have been developed to address this [241], but it is altogether abrogated by chromosomal integration strategies. Here, diverse tools are available like random chromosomal integration carried out using transposon Tn5 [242] or site-specific integration using transposon Tn7 or targeted recombination [238,243], with all methods being functional in a range of bacterial species. Of course, chromosomal integration strategies may come with constraints on flexibility compared to plasmid application.

For expression of surfactant biosynthetic genes, host-specific promoters included in the cloned expression constructs can be employed. Besides, the implementation of hybrid or synthetic promotors is a widely-used expression strategy, as recently reviewed for rhamnolipid production [110]. Most commonly applied promoters include P*_tac_* or synthetic promoters for use of *P. putida* as host [44,110,130], resistance gene promoters for expression in *B. subtilis* [119,120], and the P*_tac_* or phage promoters for *E. coli* [72,237,244]. In addition, intrinsic chromosomal promoters of the host can be readily exploited by integration in highly transcribed sites of the bacterial genome [245,246].

Expression of natural biosynthetic genes usually yields the natural product, as it does in a recombinant system. Beyond this, the quality of the final product can be optimized aiming for tailor-made “designer-molecules” [130,247]. For example, pure mono-rhamnolipids can be produced in *P. putida* by expression of genes *rhlAB* (from *P. aeruginosa* or *B. glumae*) and omitting gene *rhlC*, whereas co-expression of *rhlC* can lead to the exclusive production of di-rhamnolipids. For lipopeptide production, module engineering of the NRPS machinery, for example, modification of the adenylation domains or module deletion can lead to novel compounds as shown for polymyxin and surfactin [120,175].

Effective biosynthesis leading to high product yields not only requires a host tolerating the product but is likewise dependent on a sufficient supply of biosynthetic precursor molecules. This may be achieved by the exploitation of the intrinsic metabolism of the production hosts and by additional engineering. For rhamnolipid production as an example, the fatty acid metabolism and the carbon metabolism generating dTDP-l-rhamnose are required [215,248,249]. To enhance precursor supply, the deletion of competing pathways like polyhydroxyalkanoate biosynthesis has proven valuable [250]. Similar approaches should allow strengthening the supply of precursors for other glycolipids. For amino acid-derived non-ribosomal lipopeptides like daptomycin, an increase in production can likewise be achieved by elimination of intrinsic pathways potentially competing for precursor molecules [251].

**Figure 3:**
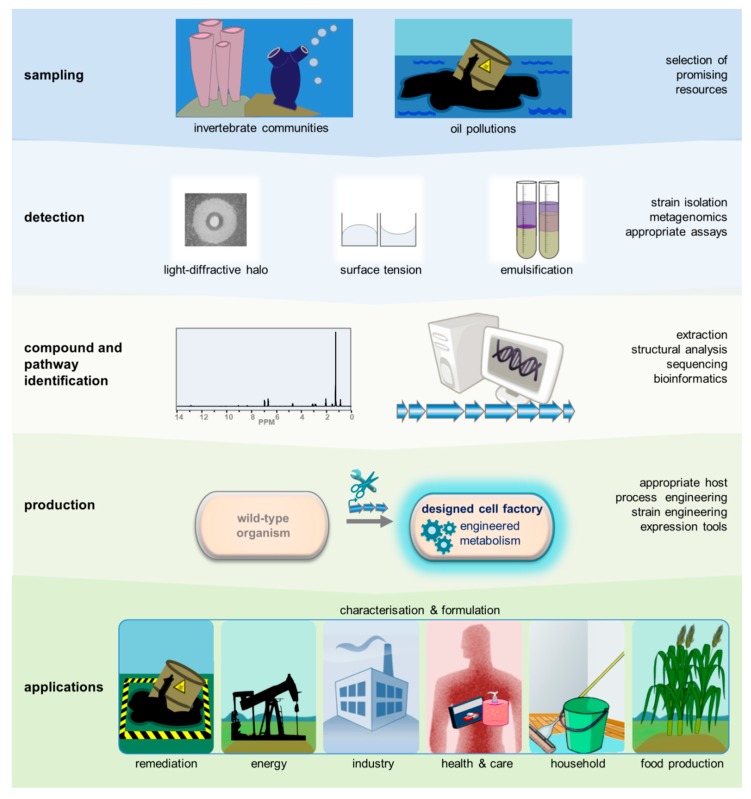
Identification, production and applications of marine biosurfactants. Production of surface-active compounds can be identified in habitats enriched for biosurfactant producers, either by culture-dependent or -independent methods using simple assays like atomised oil assay, grid assay or drop collapsing, and emulsification assay, respectively. NMR-analysis of biosurfactant structures enables the elucidation of new compounds, while sequencing and bioinformatics allows the deciphering of the biosynthetic background. Novel sophisticated strain engineering and expression tools will result in designed next-generation cell factories able to convert renewable substrates into a wealth of desired compounds with high precision and efficiency. Product yields may be further optimized by process engineering. The combination of these methods will provide biosurfactants for highly diverse applications in the future: for environmental remediation, microbially enhanced oil recovery, plant growth promotion or other applications in the food sector as well as in medical and consumer products.

Product tolerance is an intrinsic property of selected host strains with *P. putida* as a prominent example [236]. In addition, key features like an efficient export of toxic compounds, which render an organism tolerant and represent beneficial traits for high yield production, might be increased by engineering. Most biosurfactants are indeed excreted, notably both by the natural and by the recombinant producers. On the one hand, vesicles may be involved, and on the other hand, active or passive transporters may mediate product release into the medium. With respect to lipopeptide transport, studies on surfactin and arthrofactin suggest an interaction of several less-specific pumps driven by ATP- and/or proton motif force [252,253,254]. Regarding glycolipids, however, the molecular genetic basis and underlying mechanisms in bacteria are currently unknown, in contrast to the yeast system, where specific transporters have been assigned to different classes of glycolipids [255]. The identification of transporters for bacterial surfactants, in particular glycolipids, will spur the creation of next-generation production platforms with an active excretion function which may increase productivity [253,256]. In addition to molecular genetic approaches, process engineering can help to increase product yields, for example, by simultaneous separation of the biosynthetic product from the broth by *in situ* product removal. This can be achieved, for example, by adsorption or by taking advantage of the specific foaming properties of biosurfactants via foam fractionation [257,258]. Here, it becomes evident that suitable techniques from the fields of molecular genetics/bioinformatics and microbiotechnology, together with smart process development, truly synergise in the recombinant production of biosurfactants, be it from marine or other sources for a number of applications (Figure 3).

## 8. Conclusions 

Marine environments are a promising source for a large variety of surface-active metabolites. Undoubtedly, their chemical diversity is much larger than described until today and the structures of many biosurfactants still remain unknown. Furthermore, the oceans comprise a large diversity of habitats and should contain many more biosurfactant producing organisms to be discovered, in particular, in promising niches like communities associated with filter-feeding invertebrates or sites affected by toxic pollution. Here, sampling and subsequent enrichment culturing of biosurfactant producing microbes or culture-independent methods, like functional or sequence-based metagenomics, will allow retrieval of surfactant biosynthetic DNA from these niches. The availability of affordable and fast sequencing methods and bioinformatic tools for the analysis of biosynthetic pathways and metabolic networks will furthermore facilitate the elucidation of the yet largely unknown biosynthesis pathways for marine biosurfactants thereby enabling the development of synthetic biology derived concepts to construct efficient recombinant production strains. All these interdisciplinary research efforts will contribute to the identification, production and application of novel marine biosurfactants (Figure 3).

## Figures and Tables

**Table 1:** Surface-active secondary metabolites produced by marine microorganisms and the respective site of isolation.

Producing Species ^1^	Compound ^2^	Alkane-Dependent ^3^		Sampling Site ^4^	Reference
		Isolation	Production		
**fatty acids**					
*Aureobasidium pullulans*	massoia lactone			coastal seawater, Koh Sichang, Gulf of Thailand	[59]
*Cobetia* sp. strain MM1IDA2H-1	3-hydroxy fatty acids	x		eulitoral pond, Montemar, Chile	[60]
*Serratia rubidaea*	rubiwettin R1	n.a.		n.a.	[61]
**lipoamino acids**					
*Myroides* sp. SM1	ornithine lipid	x	x	seawater, Thailand	[62]
*Alcanivorax dieselolei*	proline lipid	x	x	surface water, Yellow River delta, Bohai Sea, China	[63]
*Brevibacterium luteolum*	proline lipid		x	tunicate, north coast of São Paulo, Brazil	[64]
**lipopeptides**					
*Brevibacterium luteolum*	Thr-Pro- Pro-Leu/Ile-Leu/Ile- Ala- Phe		x	tunicate, north coast of São Paulo, Brazil	[64]
*Brevibacterium aureum*	Gly-Gly-Leu-Pro		x	sponge, southwest coast of India	[65]
*Rhodococcus* sp. TW53	rhodofactin(miao)Ala-Ile-Asp-Met-Pro	x	x	deep sea sediment, Pacific Ocean	[66]
*Nocardiopsis alba*	phenyl alanine dipeptide		x	sponge, southwest coast of India	[67,68]
*Bacillus pumilus*	pumilacidin			seawater	[69,70]
*Bacillus pumilus*	surfactin-like			sponge, Hautman Reef, Australia	[71]
*Bacillus licheniformis* NIOT-06	surfactin			sponge, North Bay of Port Blair, South Andaman	[72]
*Bacillus stratophericus*	Surfactin(miao)pumilacidin	x		harbour, Sfax, Tunisia	[73]
*Bacillus* sp. CS30	surfactin			deep sea sediment, Formosa ridge, South China Sea	[74]
*Bacillus siamensis*	surfactin(miao)bacillomycin F			fish intestine, Guangzhou, China	[75]
*Bacillus licheniformis*	lichenysin	x		deep oil well, Northern Germany	[76]
*Bacillus circulans*	fengycins			marine samples, Andaman Nicobar Islands, India	[77]
*Bacillus megaterium*	iturin			seawater, Andaman Nicobar Islands, India	[78]
*Bacillus* sp. KCB14S006	iturins			saltern, Incheon, South Korea	[79]
*Bacillus amyloliquefaciens* SH-B74	plipastatin A1			deep sea, South China Sea	[80]
*Paenibacillus polymyxa*	polymyxin B			red algae	[81]
	fusaricidin B				
*Brevibacillus laterosporus*	tauramamide			tube worm, Loloata Island, Papua New Guinea	[82]
*Aneurinibacillus aneurinilyticus*	aneurinifactin	x		sea sediment, Gulf of Mannar, India	[83]
*Bacillus amyloliquefaciens*	didemnin B	x		oil-contaminated water, Red Sea, Egypt	[84]
*Tistrella mobilis*	didemnin B			seawater/tunicates, Red Sea, Pacific Ocean, and marine sediments in Japan	[85]
*Achromobacter* sp. HZ01.	Gly-Gly-Leu-Met-Leu-Leu	x		oil-contaminated water, Mabianzhou Island, southern China	[86]
*Pseudomonas* sp.	massetolide			red algae, tubeworm, Moira Island and Masset Inlet, Canada	[87,88]
*Pontibacter korlensis*	pontifactin(miao)Ser-Asp-Val-Ser-Ser	x		contaminated seawater and sediment, coastal sites of Karaikal, India	[89]
**glycolipids**					
*Pseudomonas aeruginosa*	rhamnolipid	x	x	contaminated seawater, Zhoushan Islands, China	[90]
*Pseudomonas aeruginosa*	rhamnolipid		x	coastal sediment, Odisha, India	[91]
*Pseudomonas aeruginosa*	rhamnolipid			offshore sediment, Xiamen, China	[92]
*Pseudomonas* sp. BTN-1	rhamnolipid			sediments, Baia Terranova, Antarctica	[93]
*Pseudomonas* sp. MCTG214(3b1)	rhamnolipid	x	x	coastal seawater, Sarasota Bay, Florida, US	[94]
*Buttiauxella* sp.	glucosyl ester lipid			mangrove forest, Qeshm Island, Iran	[95]
*Serratia marcescens*	glucosyl ester lipid			coral, Mandapam, India	[96]
*Serratia rubidea*	rubiwettin RG1	n.a.		n.a.	[61]
*Alcanivorax borkumensis*	glucose lipid	x	x	sediments, isle of Borkum, North Sea, Germany	[52,97,98]
*Alcanivorax*	rhamnolipid	x	x	chronically polluted harbour water, Elefsina Bay, Aegean Sea, Greece	[99]
*Paracoccus*	sophorolipid	x	x	chronically polluted harbour sediment, Elefsina Bay, Aegean Sea, Greece	
*Arthrobacter* sp. EK 1	trehalose lipid tetraester	x	x	seawater, North Sea, Germany	[49,50]
*Arthrobacter* sp. SI 1	trehalose lipid diester	x	x	seawater, North Sea, Germany	
*Rhodococcus* sp. strain PML026	trehalose lipid		x	seawater, Plymouth, UK	[100]
*Rhodococcus* sp. BS-15	tri-glucose lipid tetraester,		x	deep sea sediment, Okinawa Trough	[101]
*Actinoalloteichus hymeniacidonis*	Doktolipids(miao)(rhamnose lipids)			coastal sediment, Dokdo island, South Korea	[102]
*Streptomyces* sp. IA49E	di-rhamnolipid	x		coast with petrochemical facilities, Galveston Bay, Texas, USA	[103]
*Streptomyces* sp.CNB-253	phenazine-l quinovose ester			shallow sediments, Bodega Bay, CA, USA	[104]
*Staphylococcus lentus*	threose diester			snail, Mandapam, Tamil Nadu, India	[105]
*Cyberlindnera saturnus*	cybersan (galactose lipid)	x		polluted coastal sediment, Tamil Nadu, India	[106]

^1^ designation of the producing microorganism. ^2^ designation and/or chemical composition. ^3^ isolation indicates the producer strain was isolated from alkane/crude oil contaminated environments or from enrichment using such compounds as a sole carbon source; production indicates cultures were supplemented with hydrophobic carbon sources (x = yes). ^4^ as stated in the respective publications. n.a., data not available.

**Table 2:** Marine biosurfactants with known biosynthetic pathways.

Surface-Active Compound	Chemical Classification	Producing Bacterium	Marine Isolate ^1^	Reference
surfactin	lipopeptide	*Bacillus subtilis*	-	[33,113,114]
lichenysin	lipopeptide	*Bacillus licheniformis*	+	[115,116]
fengycins	lipopeptide	*Bacillus subtilis*	-	[117]
iturin	lipopeptide	*Bacillus subtilis*	-	[118]
plipastatin A1	lipopeptide	*Bacillus subtilis*	-	[119]
polymyxin B	lipopeptide	*Paenibacillus polymyxa*	-	[120]
fusaricidin B	lipopeptide	*Paenibacillus polymyxa*	-	[121]
didemnin B	lipopeptide	*Tistrella mobilis*	+	[85]
massetolides	lipopeptide	*Pseudomonas fluorescens*	-	[88]
rhamnolipids	glycolipid	*Pseudomonas aeruginosa*	-	[42,110]

^1^ indication if the biosynthesis was described for a marine (+) or terrestrial (-) strain.
